# Poly (ADP-Ribose) Polymerase 1 Protein Expression in Normal Pancreas and Pancreatic Adenocarcinoma

**DOI:** 10.1155/2020/2717150

**Published:** 2020-06-29

**Authors:** Roberto Castiglione, Aldo E. Calogero, Enzo Vicari, Giovanna Calabrini, Anna Cosentino, Placido D'Agati, Filippo Fraggetta, Michele Salemi

**Affiliations:** ^1^Department of Clinical and Experimental Medicine, University of Catania, Catania, Italy; ^2^Pathology Unit, Gravina Hospital, Caltagirone, Italy; ^3^Department “GF Ingrassia” Hygiene and Public Health, University of Catania, Catania, Italy; ^4^Pathology Unit, Cannizzaro Hospital, Catania, Italy; ^5^Oasi Research Institute-IRCCS, Troina, EN, Italy

## Abstract

Pancreatic cancer is a most frequent cancer in Europe, and the majority of cases of cancer of the pancreas are diagnosed above the age of 65. Radical surgery is the first curative treatment of pancreatic cancer, and alternative or combined therapeutic options, in particular, consist of adjuvant or neoadjuvant chemotherapy, with or without radiotherapy. Many factors, including diet and genetics, have been implicated in the development of cancer of the pancreas. Poly (ADP-ribose) polymerase 1 (PARP-1) protein is required for translocation of the apoptosis-inducing factor (AIF) from the mitochondria to the nucleus. It is involved in programmed cell death processes. Different PARP-1 gene expression proteins have been observed in various tumors such as lung, ovarian, endometrial, skin, and glioblastoma. We evaluated the expression of PARP-1 protein in pancreatic adenocarcinoma and normal pancreas tissues by immunohistochemistry. Protein PARP-1 in the nucleus was found in all samples (normal pancreas and pancreatic adenocarcinoma tissues). No cytoplasmic staining was observed in any sample. PARP-1-positive cells resulted higher in the normal pancreas compared with the pancreas with adenocarcinoma. PARP-1 overexpression in prostate cancer tissue compared with normal prostate suggests a greater activity of PARP-1 in these tumors. These findings suggest that PARP-1 expression in prostate cancer is an attempt to trigger apoptosis in this type of tumor, similarl to that reported in other cancers. This finding suggests that PARP-1-mediated cell death pathways are inhibited in this cancer.

## 1. Introduction

Gastrointestinal diseases are some of the most common pathologies in the European population. Some of them are characterized by acute symptoms that can lead to serious manifestations such as digestive bleeding and emergency intervention. Other pathologies, however, have a prolonged course over time and tend to become chronic [1, 2], that is, to become permanent, with a heavy impact on the quality of life. In Europe, pancreatic cancer is the sixth most prevalent cancer, accounting for 2.8% of the cancer cases among men and 3.2% among women. With a 5-year survival rate of less than 10%, it is the fifth leading cause of cancer-related deaths [3]. Ninety-four percent of the patients succumb to the disease within the first 5 years of their diagnosis [4]. The majority of the cases of pancreatic cancer are diagnosed after the age of 65, with 60% of the cases at an advanced stage, indicating little improvement in treatment and survival over the past 30 years [3]. Radical surgery offers the only curative treatment of pancreatic cancer, and alternative or combined therapeutic options, in particular, consist of adjuvant or neoadjuvant chemotherapy, with or without radiotherapy [3, 5, 6].

Many factors, including diet, habits, and genetics, have been implicated in the development of pancreatic cancer. Novel drug combinations have been reported to improve survival, advances in radiation therapy have resulted in less toxicity, and enormous strides have been made in the understanding of the fundamental genetics of pancreatic cancer [5, 6]. These advances not only offer hope but also increase the complexity of medical care. It is clear that multidisciplinary care that provides comprehensive and coordinated evaluation and treatment is the most effective way to manage pancreatic cancer [5, 6].

The poly (ADP-ribose) polymerase 1 (PARP-1) gene, located in the chromosome region 1q42, is 43 kb long and is composed of 23 exons (OMIM 173870). The PARP-1 function may contribute to efficient maintenance of genome integrity [6]. PARP-1 activation is required for the translocation of the apoptosis-inducing factor (AIF) from the mitochondria to the nucleus. The PARP-1 protein is then proteolytically cleaved at the onset of apoptosis by the caspase 3 enzyme [7]. Furthermore, PARP-1 activity and the poly (ADP-ribose) [PAR] polymer mediate PARP-1-induced cell death [7, 8]. *In vivo* pharmacological and genetic studies have revealed that overexpression of PARP-1 is the key mediator of programmed necrotic cell death. In addition, PARP-1 appears to be involved in other programmed cell death processes, such as macroautophagocytic cell death and apoptosis [9]. Microarray analysis of PARP-1 gene expression in more than 8,000 samples has revealed that PARP-1 is highly expressed in several types of tumors compared with the corresponding healthy tissues. Furthermore, PARP-1 mRNA expression in a wide variety of histologically normal tissues is low and uniform [10]. Overall, the most striking differences in PARP-1 expression have been observed in breast, ovarian, endometrial, lung, and skin cancers and in non-Hodgkin's lymphoma [10].

The present study was undertaken to immunohistochemically evaluate and compare the expression of the PARP-1 protein between healthy pancreatic tissue samples and pancreatic adenocarcinoma (PA) tissue samples.

## 2. Materials and Methods

### 2.1. Patients and Controls

A comparison was made between seven healthy pancreatic tissue samples obtained during postmortem examination of donors without cancer (four men and three women) and five PA tissues stained with haematoxylin and eosin. At least two operators evaluated the tissue sections. The protocol was approved by the internal Institutional Review Board, and written informed consent was obtained from each PA patient and control donor or, if deceased, from his/her relatives.

### 2.2. Immunohistochemical Staining

Pancreatic-tissue sections of 4 *μ*m thickness were obtained from all control donors and PA patients. All the sections were formalin-fixed and paraffin-embedded by standard methods. PARP-(F-2), a mouse monoclonal antibody raised against the PARP-1 protein, was used for the immunohistochemical analysis (Santa Cruz Biotechnology, Inc., Heidelberg, Germany; PARP-1 [F-2]: sc-8007). As indicated in the manufacturer's instructions, this antibody, at a dilution of 1 : 300, is known to reliably recognize PARP-1 proteins in PA and healthy tissues in an immunohistochemical analysis. The slides were deparaffinized, rehydrated, subjected to three 5 min cycles in a microwave at 360 W in a citrate buffer, preincubated in citrate buffer with 3% H_2_O_2_, and thoroughly washed in Tris-buffered saline (TBS) (50 mM Tris-Cl, 150 mM NaCl, pH 7.4) containing 0.05% of Tween 20 (washing buffer). The slides were then preincubated for 30 min in TBS with 3% of bovine serum albumin and, then, with the anti-PARP-1 antibody (1 : 300) in TBS containing 1% of BSA. The slides were then thoroughly washed with a washing buffer, and specific signals were visualized with the LSAB 2 kit (anti-mouse, biotinylated, and peroxidase-labelled streptavidin) and 3,3′-diaminobenzidine-tetrahydrochloride (DAB·4HCl; Dako, Carpinteria CA, USA). After detection, the tissue sections were counterstained with haematoxylin, dehydrated, and mounted in a xylene-based DPX mountant (BDH, Poole, UK) [11].

### 2.3. Microscopic Evaluation

Slides were examined, and the cells were visually scored at 20X and 40X magnification. To evaluate the percentage of signal-positive tumor cells, microscopic visual fields were chosen such that nontumorous cells were also included. The percentage of PARP-1-positive cells was evaluated independently in a blinded fashion by two co-authors (M.S. and M.A.). No significant difference was found between their observations.

### 2.4. Statistical Analysis

**Figure 1:**
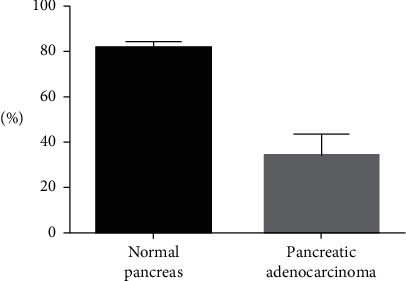
T-test data (mean with SEM).

Results are expressed as mean ± SEM. Data were analyzed by the t-test, and statistical analysis was performed in GraphPad Prism 5 (GraphPad Software, Inc. La Jolla, CA, USA). Statistical significance was assumed at *p* values lower than 0.05 (Figure 1).

## 3. Results

**Figure 2:**
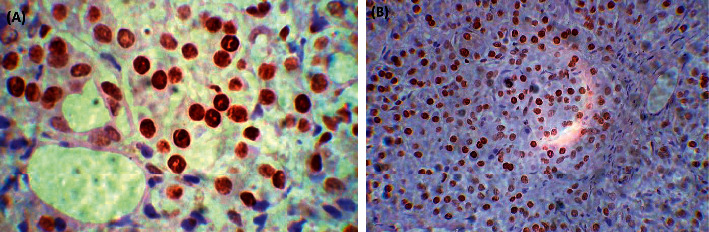
(a) Immunohistochemistry of pancreatic adenocarcinoma scored at 40x. Hematoxylin counterstain; (b) immunohistochemistry of normal pancreas scored at 20x. Hematoxylin counterstain.

Expression of the PARP-1 protein was evaluated in five PA tissue samples. A PARP-1-positive nuclear signal was present in all five samples with a 34% ± 9.5% of nuclei involved (mean ± SEM; Table 1; Figure 2(a)). No cytoplasmic staining was observed in any samples. Healthy pancreatic tissues (seven samples of postmortem tissue) yielded a positive nuclear signal for protein PARP-1 (Table 1; Figure 2(b)) with 81.42% ± 3% of the nuclei involved, whereas no cytoplasmic staining was observed in any of the healthy-tissue samples. The signal was present on the nuclear membrane and in nuclear chromatin in all the samples (healthy and PA tissues). In this study, the percentage of PARP-1-positive cells was lower in tumors of patients with PA than in control tissue samples.

## 4. Discussion

We found that, in comparison with the controls, the PARP-1 protein was downregulated in PA tissue samples (Table 1). This finding suggests that PARP-1-mediated apoptotic pathways are inhibited in pancreatic cancer. These results support the hypothesis that, in some tumors (e.g., melanoma), apoptotic pathways are inhibited at different levels [12].

These results contradict the results of other studies on the expression of the PARP-1 protein in prostate carcinoma [13], glioblastoma [14], squamous cell carcinoma [15], non-Hodgkin's lymphoma, and other cancers such as breast, ovarian, lung, endometrial, and skin malignant tumors [16]. PARP-1 is an abundant, chromatin-associated enzyme present in all eukaryotic cell nuclei, where it plays an important role in the maintenance of genomic integrity and transcriptional control [16,17]. A poly (ADP-ribose) polymerase 1 inhibitor increases an antitumor activity against glioma, intracranial melanoma, lymphoma, and haematological neoplasia [18–21]. Activation of the PARP-1 gene in response to DNA damage is an important mechanism of homeostasis or of triggering apoptosis. Our preliminary data suggest that, in a healthy pancreas, almost 80% of the nuclei express the PARP-1 protein; this level is enough for its proapoptotic activity. In contrast, the PARP-1 protein is expressed to a lesser extent in PA tissues, suggesting that the proapoptotic activity of PARP-1 is lower and that the mechanisms of DNA repair by PARP-1 are less active in PA. These data may partially explain the aggressiveness of PA compared to other cancers. In conclusion, if these results can be reproduced in a larger cohort, then PARP-1 expression can serve as a marker for the differential diagnosis between PA and other pancreatic disorders.

## Figures and Tables

**Table 1:** Characteristics of healthy pancreas tissue donors and of patients with pancreatic adenocarcinoma.

Normal pancreas (autopsy)				Pancreatic adenocarcinoma						
ID	Sex	Age	Positive (cell nuclei %)	ID	Sex	Age	Positive (cell nuclei %)	pT	N	G
1	M	82	75	1	M	90	10	T2	N0	G2
2	F	95	80	2	F	62	55	T2	N0	G2
3	M	94	85	3	M	72	15	T2	N0	G2
4	F	92	85	4	M	61	55	T2	N1	G2
5	M	75	70	5	M	84	35	T2	N0	G2
6	M	78	95							
7	F	84	80							

Mean			81.4				34			
SD			8.0				21.3			
SEM ±			3.0				9.5			

ID: identification; pT: tumor stage; N: lymphonodes; G: grading; T-test = 5.4539 (*p* < 0.005); *p* value = 0.0003(^*∗∗∗*^).
